# In vivo evaluation of vulvar lichen sclerosus with reflectance confocal microscopy and therapeutic monitoring in children

**DOI:** 10.1111/srt.13234

**Published:** 2022-11-15

**Authors:** Lixin Chen, Ying Wang, Xibo Gao, Bei Qin, Min Ren, Wanxing Zhang, Ran Wei, Haihui Su, Qinfeng Li

**Affiliations:** ^1^ Department of Dermatology Tianjin Children's Hospital Tianjin China

**Keywords:** children, histopathology, lichen sclerosus, reflectance confocal microscopy, therapeutic monitoring, vulvar

## Abstract

**Background:**

Vulvar lichen sclerosus (VLS) in girls presents with itching, dysuria, and constipation and may result in the loss of vulvar architecture. In patients with an ambiguous clinical presentation, reflectance confocal microscopy (RCM) could be a helpful noninvasive diagnostic tool. The aim of this study was to describe the RCM characteristics of VLS and explore the clinical application value of RCM in therapeutic monitoring.

**Methods:**

Sixteen patients with VLS were included in the study. All patients were periodically evaluated clinically with RCM, and different treatment regimens were given based on the patient's clinical appearances and RCM features.

**Results:**

Some major key diagnostic features of VLS can be observed by RCM, including round to oval cyst‐like structures with medium‐to‐low‐refractive keratinoid substances (75%), thinning of the epidermal thickness (100%), destruction of the ring‐like structures around dermal papillae (100%), disorderly distributed coarse medium‐refractive fibrous material (100%),polygonal, plump, high‐refractive cellular structures and linear low‐refractive canalicular structures (100%). All of these characteristics had a high correspondence with histopathological features. The clinical manifestations improved after individualized treatment regimens based on the clinical appearances and RCM features.

**Conclusion:**

RCM allows the visualization of major key diagnostic features of VLS and represents a valid option for objective therapeutic monitoring.

## 1 INTRODUCTION

Vulvar lichen sclerosus (VLS) is a chronic inflammatory dermatosis with unclear etiology and pathogenesis characterized by porcelain‐white atrophic plaques around the vulvar and anal areas, resulting in scarring, skin atrophy, and dysfunction. VLS is associated with genital squamous cell carcinoma, necessitating early diagnosis, timely treatment and long‐term follow‐up.^1^ Reflectance confocal microscopy (RCM) can noninvasively observe changes in skin cell levels, which are highly consistent with histopathology.^2^, ^3^ In this study, preliminarily described the use of RCM for evaluating the characteristic features and conducting therapeutic monitoring of VLS in children.

## 2 MATERIALS AND METHODS

### 2.1 Patients

We performed this study on 16 patients with histological and RCM confirmation of VLS admitted to our department between September 2016 and May 2021. All patients were aged 4–11 years. The enrolled patients had neither received any treatment nor had been diagnosed with other vulvar dermatoses. The skin symptoms had persisted for 3 months to 1 year. All the patients were female and did not have any extragenital involvement.

### 2.2 Methods

All patients included in the study were examined using an available RCM system, the VivaScope 1500 (Lucid Inc., USA). The system uses an infrared 830‐nm diode laser operating at a power of less than 20 mW at the tissue level. A ×30 water‐immersion objective lens with a numerical aperture of 0.9 was used, and pure water (refractive index = 1.33) was used as an immersion medium. The probe was routinely disinfected with 75% alcohol before and after each examination.

All patients were periodically evaluated clinically and for RCM features. Individualized treatment regimens were given based on clinical appearances and RCM features. (1) Mometasone furoate 0.1% ointment was given twice a day for the active phase of the sclerotic stage, and the patients were reevaluated every 3 weeks. (2) For the convalescence phase, Mometasone furoate 0.1% ointment was given once a day in the morning, and topical calcineurin inhibitors (tacrolimus 0.03% ointment) were given once a day in the evening, we reevaluated the patients every 6 weeks. (3) For the maintenance phase, tacrolimus 0.03% ointment was administered every other day, and mucopolysaccharide polysulfate cream was administered twice daily, the patients were reevaluated every 8 weeks.

## 3 RESULTS

### 3.1 CLINICAL FEATURES

Among the 16 children enrolled, 10 had itching (10/16, 62.5%), 3 had constipation (3/16, 18.8%), and 6 were asymptomatic (6/16, 37.5%). We detected clearly demarcated porcelain‐white atrophic plaques (16/16, 100%), telangiectasia (9/16, 56.3%), erosions (4/16, 25%) and ecchymosis (4/16, 25%).

### 3.2 HISTOPATHOLOGY IMAGES

**FIGURE 1:**
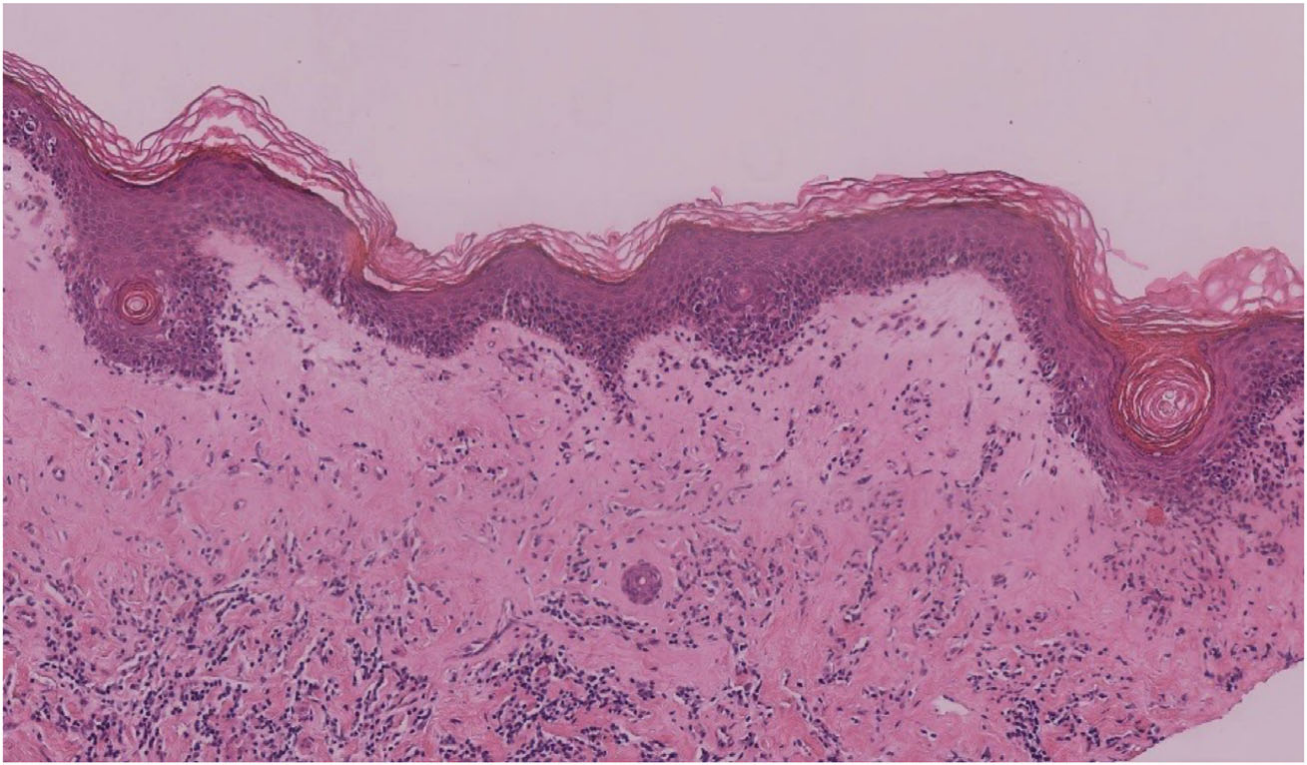
Histopathology images of VLS Hyperkeratosis, follicular plugs, epidermal atrophy, liquefaction degeneration of basal cells, and the homogenization of collagen in the superficial dermis, and an infiltrate of lymphocytes and melanophages

Hyperkeratosis, follicular plugs, epidermal atrophy, liquefaction degeneration of basal cells, and a band of homogenization of collagen in the superficial dermis were observed, and there was an infiltrate of dense lymphocytes and melanophages (Figure 1).

### 3.3 RCM CHARACTERISTICS

The features of VLS RCM images were analyzed at different stages and from the epidermis to the dermis (Table 1).

**TABLE 1:** Features of RCM images at different phases of VLS

Features of RCM images		Sclerotic stage	Convalescence phase	Maintenance phase
Epidermis	Hyperkeratosis	31.3%	(−)	(−)
	Cyst‐like structures	75%	25%	(−)
	Epidermal thickness	Obvious thinning	Slight atrophy	Normal
Dermoepidermal junction/dermal papillary rings		Destruction	Irregular ring‐like structure	Ring‐like structure
Dermis	Fibrous material	(++)	(±)	(−)
	Polygonal cellular structures	(++)	(±)	(−)
	Canalicular structures	(++)	(±)	(−)
	Others	‐	Particulate‐matter structures (+)	Homogeneously round cells (+)

**FIGURE 2:**
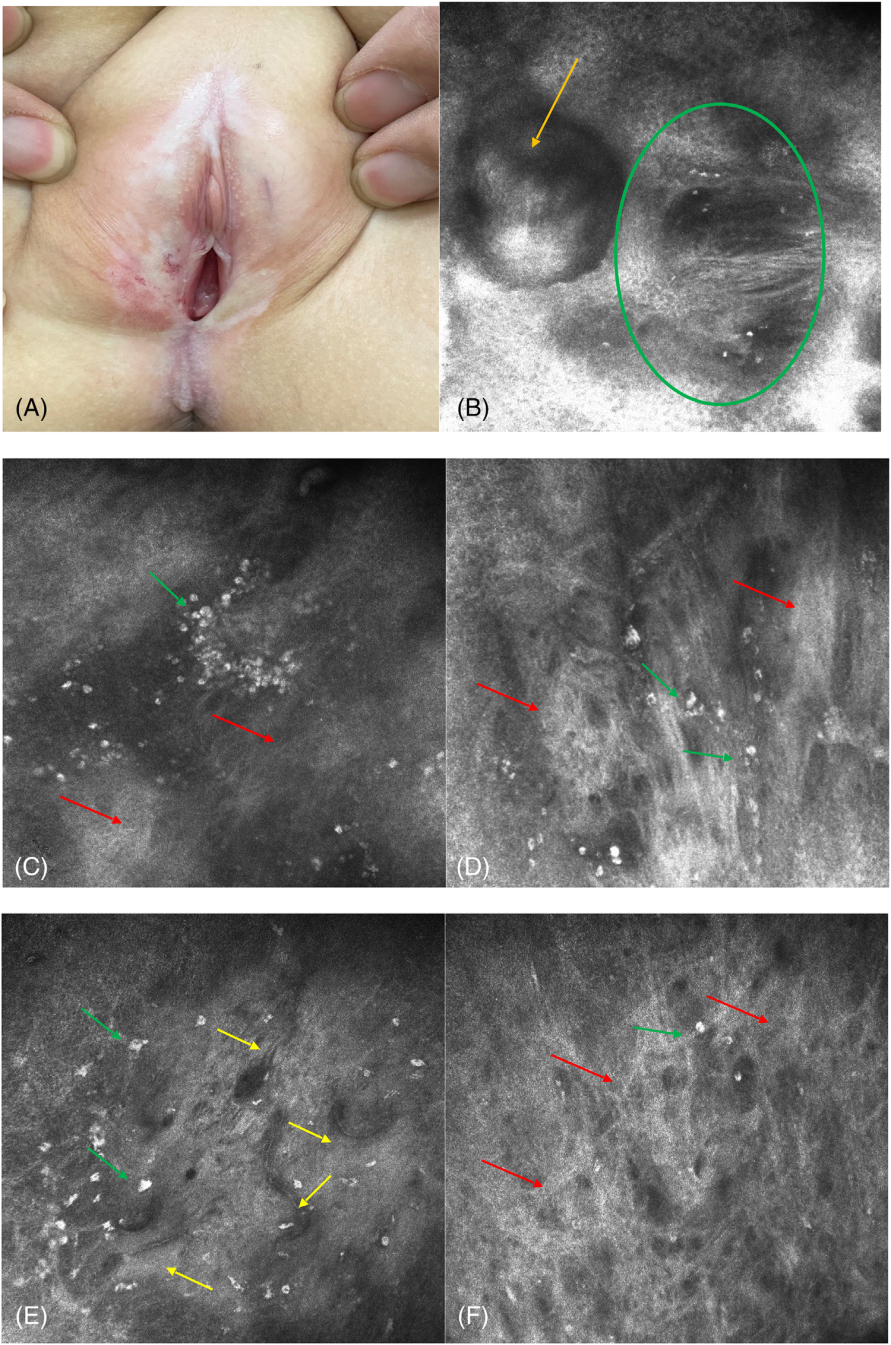
Clinical presentation and key RCM features of VLS in the sclerotic stage Clearly demarcated porcelain‐white atrophic plaques, telangiectasia and ecchymosis can be seen (2a). RCM examination shows round to oval cyst‐like structures containing medium‐low‐refractive keratinoid amorphous substances (orange arrow); destruction of the dermal papillary rings (green oval); white, coarse, diffuse and disorganized hyperrefractive fibrous material, and some of the material in fascicular aggregates (red arrow); polygonal, plump, highly refractive cellular structures (green arrow); an increased number of low‐refractive canalicular structures filled with high‐refractive flow blood cells and horizontally arranged on confocal sections (yellow arrow)

In the active phase of the sclerotic stage, in the epidermis, we detected hyperkeratosis (5/16, 31.3%), round to oval cyst‐like structures containing medium‐low‐refractive keratinoid amorphous substances (12/16, 75%), and thinning of the epidermal thickness (16/16, 100%). At the dermoepidermal junction, inflammatory involvement and destruction of the dermal papillary rings were detected, and they appeared nonedged and nonrimmed (16/16, 100%). There was substantial white, coarse medium‐refractive fibrous material that appeared diffuse and disorganized, and some of the material gathered in fascicular aggregates in the upper dermis (16/16, 100%). Uniformly round cells with high refraction and many polygonal, plump, oval to stellar in shape, highly refractive cellular structures were aggregated (16/16, 100%). There were also many low‐refractive linear or canalicular structures filled with high‐refractive flow peripheral blood cells that were horizontally arranged on the confocal sections (16/16, 100%) (Figure 2).

**FIGURE 3:**
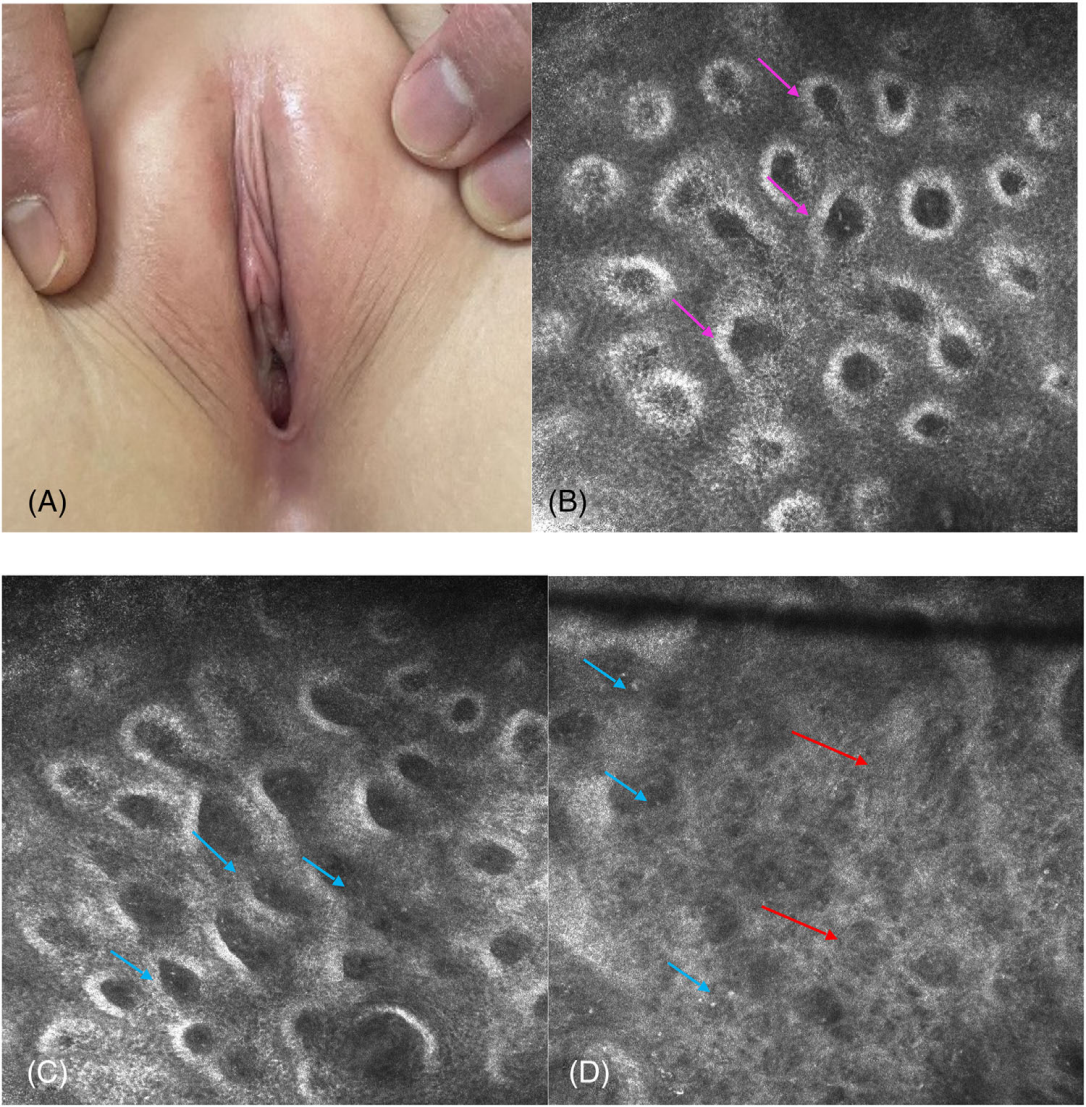
Clinical presentation and key RCM features of VLS in the convalescence phase Skin texture and color returned to normal (3a). RCM examination shows an irregular ring‐like structure in the dermoepidermal junction (pink arrow), and fibrous material (red arrow) was significantly reduced. Numerous particulate matter structures were detected in the dermis (blue arrow)

At the convalescence phase, the epidermal examination revealed slight atrophy, no hyperkeratosis (16/16, 100%) and obvious decreases in cyst‐like structures (4/16, 25%). Irregular ring‐like structures with uneven refraction were visible at the dermoepidermal junction (13/16, 81.3%). There were significantly fewer fibrous materials, polygonal structures and canalicular structures (14/16, 87.5%) and numerous particulate matter structures with medium‐high refractive diffused distributions in the dermis (16/16, 100%) (Figure 3).

**FIGURE 4:**
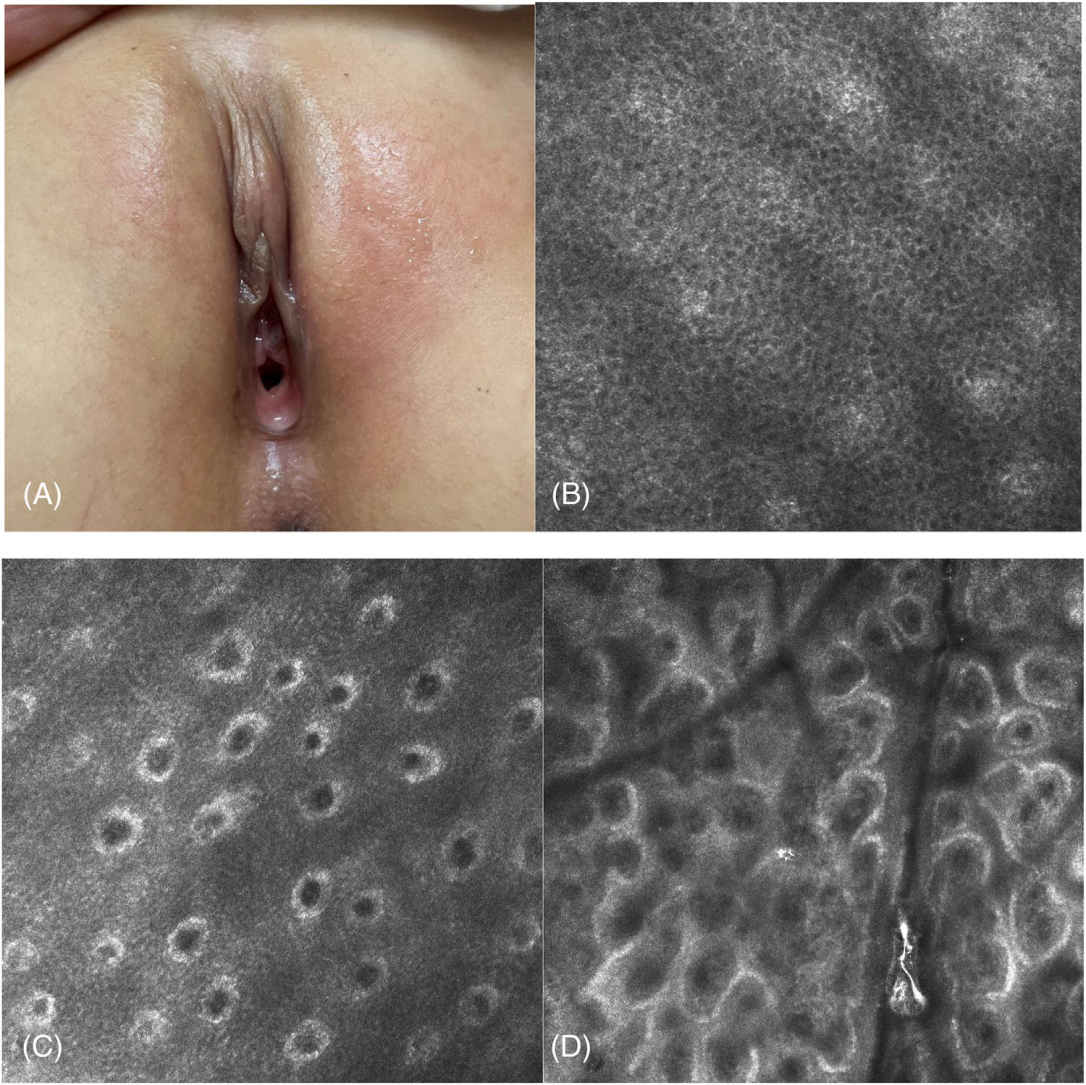
Clinical presentation and key RCM features of VLS in the maintenance phase There are no obvious abnormalities in the lesion area (4a). RCM examination revealed a normal honeycomb pattern of epidermal and ring‐like structures (4b, 4c). Homogeneously round cells can be seen in the upper dermis

During the maintenance phase, the epidermal examination revealed a normal honeycomb pattern (16/16, 100%), and a ring‐like structure with uniform high refractive was observed in the dermoepidermal junction (16/16, 100%). Small, homogeneously round cells with high refraction could be seen in the upper dermis (13/16, 81.3%), and there was no fibrous material, polygonal structure or canalicular structures (Figure 4).

## 4 DISCUSSION

VLS is a chronic inflammatory dermatosis of unclear etiology, and its prevalence in the whole population is essentially unknown.^4^ VLS presents a bimodal distribution, occurring in childhood and in postmenopausal women.^5^ It was previously believed that pediatric VLS resolves at or after puberty; however, recent evidence has shown that while there may be improvement, true remission cannot be assumed.^4^ One study has shown that at least 75% of girls still have ongoing symptoms and signs,^6^ suggesting that children with VLS require a correct diagnosis, active management and long‐term follow‐up.^7^, ^8^ Pediatric patients are a special group in whom invasive procedures such as biopsies are not always possible. RCM is a real‐time, repeatable, adjuvant diagnostic tool with a resolution comparable to histopathology.^2^, ^3^ Recent reports have described the RCM features of lichen sclerosus in adults;^9^, ^10^, ^11^ however, there have been few studies on the diagnosis, therapeutic monitoring, and follow‐up of VLS by RCM in children.^12^

We sought to clarify the main identifying characteristics of VLS by evaluating the epidermis, the dermo‐epidermal junction and the dermis with RCM and found a high corresponding relationship between the RCM images and histopathological features (Table 2). The round to oval cyst‐like structures with medium‐low‐refractive keratinoid substances in the epidermis corresponded to the histopathological follicular plugs. The thickness of the epidermis was thinner than that of the normal skin, which corresponded to epidermal atrophy. At the dermo‐epidermal junction, the destruction of the ring‐like structures around the dermal papillae corresponded to pathological liquefaction degeneration of basal cells. The disorderly, distributed, coarse medium‐refractive fibrous material at the superficial dermis corresponded to the homogenization of collagen. The polygonal, high‐refractive cellular structures corresponded to melanophages. The linear low‐refractive canalicular structures, filled with flow blood cells, corresponded to dilated blood vessels on histopathology. This suggests that RCM can reveal major key diagnostic features of VLS.

**TABLE 2:** RCM/histopathological features of VLS

	RCM features	Histopathological features
Epidermis	Round to oval cyst‐like structures containing medium‐low‐refractive keratinoids amorphous substance	Follicular plugs
	Thinning of the epidermal thickness	Epidermal atrophy
Dermoepidermal junction	An inflammatory involvement and destruction of the dermal papillary rings	Basal cell degeneration
Dermis	Disorganized distributed white, coarse medium‐refractive fibrous material	Homogenization of collagen
	Polygonal, plump, oval to stellar in shape, high‐refractive cellular	Melanophages
	Low‐refractive linear or canalicular structures, filled with high‐refractive flow peripheral blood cells	Dilated blood vessels

Therapeutically, it is now accepted that ultrapotent or potent topical corticosteroids (TCS) should be used as the first‐line treatment for VLS,^13^, ^14^, ^15^ and some scholars believe that calcineurin inhibitors are an effective alternative to TCS in the treatment of VLS.^15^, ^16^ The treatment regimen was adjusted solely according to the condition of the skin lesion, and some studies have suggested a grade of hyperkeratosis as a guide to the choice of topical therapy.^1^, ^7^ Treatment was initiated with superpotent or potent TCS when the patient had thick white hyperkeratosis. When the lesions were controlled and all of the signs and symptoms were in remission, the potency of the TCS was slowly titrated down to a moderate‐mild potency. However, to date, no study has resulted in a universally accepted severity grade, which requires objective and accurate therapeutic evaluation criteria. In this study, different treatment regimens were given based on the skin lesions and RCM characteristics. At the active phase of the sclerotic stage, we detected clearly demarcated white atrophic plaques and thinning of the epidermal thickness, the destruction of dermal papillary rings, disorganized, distributed medium‐refractive fibrous materials, and polygonal cellular structures and canalicular structures with RCM. At this stage, mometasone furoate 0.1% ointment was given twice daily, and the patients were reevaluated every 3 weeks. When the patient was in the convalescence phase, the skin texture and color had returned to normal, and according to RCM, the epidermal examination revealed slight atrophy and an irregular ring‐like structure in the dermoepidermal junction. The fibrous material, polygonal structure and canalicular structures were significantly less obvious than before. Numerous particulate matter structures could be detected in the dermis. We adjusted the treatment regimen with mometasone furoate 0.1% ointment once a day in the morning and topical tacrolimus 0.03% ointment once a day in the evening, and we reevaluated the patients every 6 weeks. The maintenance regimens were associated with no obvious abnormalities in the lesion area. RCM examination revealed a normal honeycomb pattern of epidermal and ring‐like structures in the dermoepidermal junction. Homogeneously round cells could be seen in the upper dermis, and there was no fibrous material, polygonal structures or canalicular structures. At this stage, tacrolimus 0.03% ointment was administered every second day, mucopolysaccharide polysulfate cream was administered twice daily, and the patients were reevaluated every 8 weeks. All patients were followed up with 28 weeks, and there was no recurrence of symptoms or side effects, such as atrophy or scarring. When therapeutic monitoring was continued for 56 weeks, 5 patients ceased the treatment regimens, and 2 had experienced symptom recurrence. Many studies have emphasized that patients with VLS must establish long‐term, effective maintenance regimens to prevent disease recurrence and remote effects.^4^, ^7^, ^14^ Our findings are consistent with those in previous studies, and we recommend RCM as a complementary tool for therapeutic monitoring.

In summary, RCM allows the visualization of major key diagnostic features of VLS and represents a valid option for therapeutic monitoring. The value of RCM in VLS needs further evaluation, but it appears to be a useful adjuvant tool for diagnosis and therapeutic monitoring.

## CONFLICT OF INTEREST

None.

## Data Availability

The data that support the findings of this study are available from the corresponding author upon reasonable request.
